# Supplementary material for: Mechanisms governing the pioneering and redistribution capabilities of the non-classical pioneer PU.1
Source: Nat Commun. 2020 Jan 21;11:402. doi: 10.1038/s41467-019-13960-2 (PMC6972792; doi:10.1038/s41467-019-13960-2)
Supplement: Supplementary file 7 — Source data [file 41467_2019_13960_MOESM7_ESM.zip › Source_Data/FigureS5/FigureS5B_MotifsETS1specificPeaks/homerResults/motif4.similar.html]

motif4

## Information for motif4

A
G
T
C
C
T
G
A
C
T
G
A
G
A
C
T
A
C
G
T
A
T
G
C
A
G
T
C
A
G
T
C
C
G
T
A
T
A
C
G
  
Reverse Opposite:  

A
T
G
C
G
C
A
T
T
C
A
G
T
C
A
G
T
A
C
G
T
G
C
A
C
T
G
A
G
A
C
T
G
A
C
T
T
C
A
G
  

|  |  |
| --- | --- |
| p-value: | 1e-95 |
| log p-value: | -2.194e+02 |
| Information Content per bp: | 1.655 |
| Number of Target Sequences with motif | 315.0 |
| Percentage of Target Sequences with motif | 27.75% |
| Number of Background Sequences with motif | 487.4 |
| Percentage of Background Sequences with motif | 5.97% |
| Average Position of motif in Targets | 102.7 +/- 50.1bp |
| Average Position of motif in Background | 99.5 +/- 67.4bp |
| Strand Bias (log2 ratio + to - strand density) | -0.3 |
| Multiplicity (# of sites on avg that occur together) | 1.09 |
| Motif File: | file (matrix) reverse opposite |

### Similar de novo motifs found

|  |  |  |  |  |  |  |  |
| --- | --- | --- | --- | --- | --- | --- | --- |
| Rank | Match Score | Redundant Motif | P-value | log P-value | % of Targets | % of Background | Motif file |
| 1 | 0.833 | C G T A G A C T A C G T G T A C G A T C G T A C C T G A C T A G | 1e-48 | -111.281364 | 21.76% | 6.86% | motif file (matrix) |
| 2 | 0.809 | G A C T A C G T A G T C A G T C A G T C C G T A A C T G | 1e-35 | -82.095923 | 36.04% | 18.87% | motif file (matrix) |
| 3 | 0.786 | A G C T C G A T A T G C A G T C A T G C G C T A A T C G C G T A | 1e-35 | -82.006836 | 23.96% | 9.93% | motif file (matrix) |
